# Hair Cell Loss, Spiral Ganglion Degeneration, and Progressive Sensorineural Hearing Loss in Mice with Targeted Deletion of *Slc44a2/Ctl2*

**DOI:** 10.1007/s10162-015-0547-3

**Published:** 2015-10-13

**Authors:** Pavan Kommareddi, Thankam Nair, Bala Naveen Kakaraparthi, Maria M. Galano, Danielle Miller, Irina Laczkovich, Trey Thomas, Lillian Lu, Kelli Rule, Lisa Kabara, Ariane Kanicki, Elizabeth D. Hughes, Julie M. Jones, Mark Hoenerhoff, Susan G. Fisher, Richard A. Altschuler, David Dolan, David C. Kohrman, Thomas L. Saunders, Thomas E. Carey

**Affiliations:** Kresge Hearing Research Institute, Department of Otolaryngology/Head and Neck Surgery, University of Michigan, 1150 West Medical Center Drive, 5311 Medical Science I, Ann Arbor, MI 48109-5616 USA; Unit for Laboratory Animal Medicine, University of Michigan, Ann Arbor, MI 48109 USA; Department of Internal Medicine, Division of Molecular Medicine and Genetics, University of Michigan, Ann Arbor, MI 48109 USA; Biomedical Research Core Facilities, Transgenic Animal Model Core, University of Michigan, Ann Arbor, MI 48109 USA; Department of Human Genetics, University of Michigan, Ann Arbor, MI 48109 USA; Department of Clinical Sciences and Temple Clinical Research Institute, Temple University School of Medicine, Philadelphia, PA USA

**Keywords:** choline transporter-like protein 2, solute carrier protein 44a2, supporting cells, auditory brain stem responses, hair cell loss, spiral ganglion cell loss, murine gene knockout

## Abstract

SLC44A2 (solute carrier 44a2), also known as CTL2 (choline transporter-like protein 2), is expressed in many supporting cell types in the cochlea and is implicated in hair cell survival and antibody-induced hearing loss. In mice with the mixed C57BL/6-129 background, homozygous deletion of *Slc44a2* exons 3–10 (*Slc44a2*^Δ/Δ^) resulted in high-frequency hearing loss and hair cell death. To reduce effects associated with age-related hearing loss (ARHL) in these strains, mice carrying the *Slc44a2*^Δ^ allele were backcrossed to the ARHL-resistant FVB/NJ strain and evaluated after backcross seven (N7) (99 % FVB). *Slc44a2*^Δ/Δ^ mice produced abnormally spliced *Slc44a2* transcripts that contain a frameshift and premature stop codons. Neither full-length SLC44A2 nor a putative truncated protein could be detected in *Slc44a2*^Δ/Δ^ mice, suggesting a likely null allele. Auditory brain stem responses (ABRs) of mice carrying the *Slc44a2*^Δ^ allele on an FVB/NJ genetic background were tested longitudinally between the ages of 2 and 10 months. By 6 months of age, *Slc44a2*^Δ/Δ^ mice exhibited hearing loss at 32 kHz, but at 12 and 24 kHz had sound thresholds similar to those of wild-type *Slc44a2*^+/+^ and heterozygous *+/Slc44a2*^Δ^ mice. After 6 months of age, *Slc44a2*^Δ/Δ^ mutants exhibited progressive hearing loss at all frequencies and *+/Slc44a2*^Δ^ mice exhibited moderate threshold elevations at high frequency. Histologic evaluation of *Slc44a2*^Δ/Δ^ mice revealed extensive hair cell and spiral ganglion cell loss, especially in the basal turn of the cochlea. We conclude that *Slc44a2* function is required for long-term hair cell survival and maintenance of hearing.

## Introduction

SLC44A2 (solute carrier 44a2) is a transmembrane glycoprotein (Nair et al. 2004) that belongs to the solute carrier family of osmolyte transporters involved in transmembrane transport of small molecules (He et al. 2009). It is highly expressed in inner ear supporting cells and was discovered as the target of a monoclonal antibody, KHRI-3 (Zajic et al. 1991). In vivo administration of KHRI-3 resulted in hearing loss in mice and guinea pigs, thereby implicating this protein in antibody-mediated hearing loss (Nair et al. 1995, 1997, 1999). SLC44A2 is also strongly expressed in supporting cells in the human inner ear and is involved in autoimmune hearing loss in humans (Disher et al. 1997; Zeitoun et al. 2005; Kommareddi et al. 2009). Since antibody to SLC44A2 adversely affects hair cell survival and hearing, we postulated that this protein must be essential for normal function of the inner ear. To study its role in hearing, we created targeted knockout mice that carried a deletion of exons 3–10 of the *Slc44a2* gene. Initial studies of the effects of this deletion on a C57BL/6 genetic background revealed hair cell loss and hearing loss, but were hampered by the age-related hearing loss (ARHL) associated with the *Cdh23*^*753A*^ variant carried by C57BL/6J (Noben-Trauth et al. 2003). We backcrossed the *Slc44a2* deletion onto the FVB/NJ strain, which carries the ARHL-resistant *Cdh23*^*753G*^ or wild-type allele. Here, we demonstrate that the *Slc44a2* deletion on a majority FVB background results in early-onset hearing loss, particularly at high frequency. The hearing loss is progressive and is associated with extensive hair cell and spiral ganglion cell loss. Thus, this study indicates a critical role of *Slc44a2* in the maintenance of auditory function.

## Methods

### Targeting Vector Construction and Targeted Disruption of the *Slc44a2* Gene in Embryonic Stem Cells

**FIG. 1:**
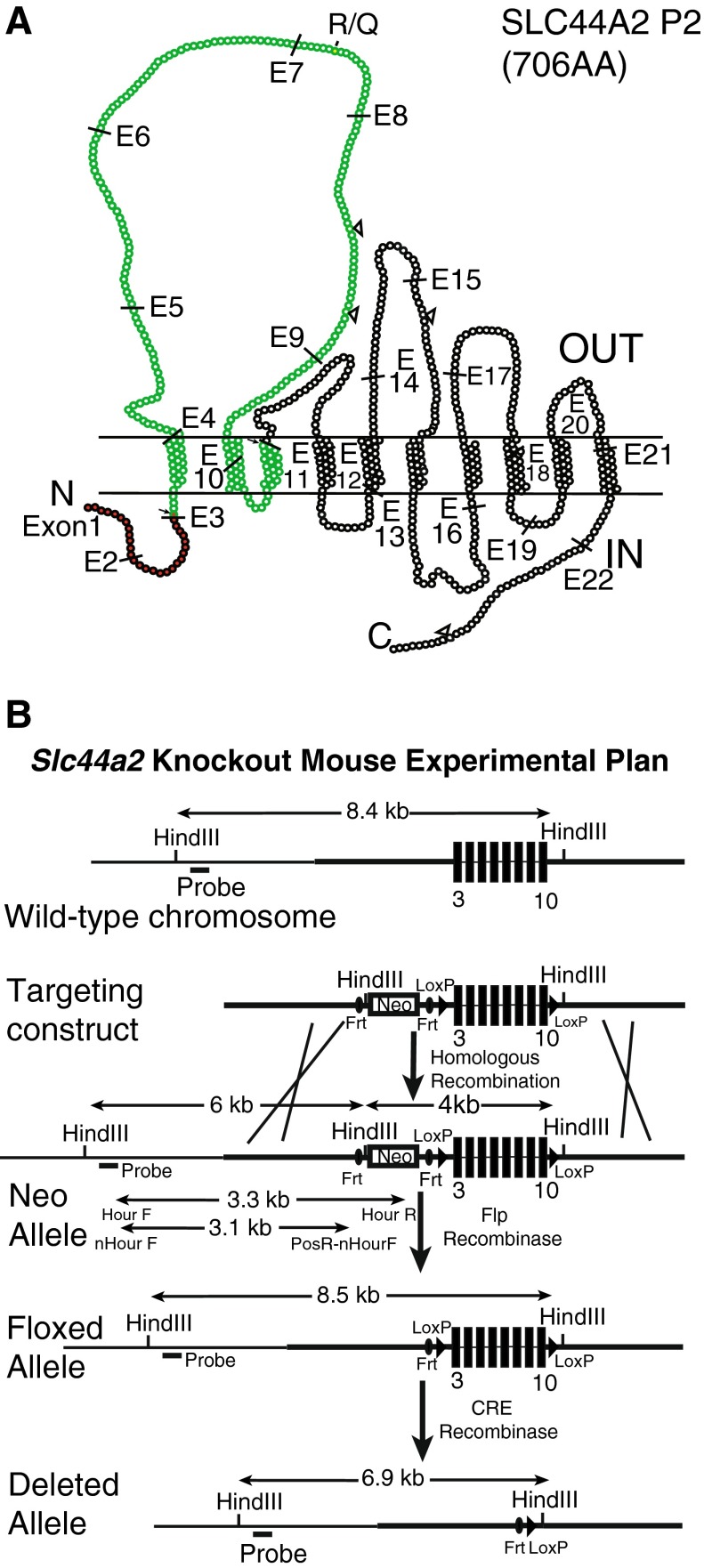
**A** The predicted topology of the SLC44A2 isoform P2 protein. Each *ball* represents an individual amino acid. The region targeted for deletion in the knockout is shown in *green*. The amino acids colored *red* are included in the first 29 amino acids encoded by a truncated and aberrantly spliced *Slc44a2* mRNA produced in the knockout mouse. *Small arrows* at E3 and E11 point to the sites where the aberrant transcript is spliced, leading to an out-of-frame transcript that is terminated by two stop codons in exon 14. *Open triangles* indicate locations of predicted N-linked glycosylation sites. *R/Q* indicates the codon 152 (isoform P1)/154 (isoform P2) arginine/glutamine polymorphism. **B** Experimental design of the *Slc44a2* gene-knockout mouse. Exons 3–10 of the mouse *Slc44a2* gene were chosen for deletion. The targeting construct was created with a 5′ homology region followed by a neo-cassette flanked by FRT sites, loxP sites flanking exons 3–10, and finally, the 3′ homology region. Mice carrying the targeting construct in the correct location were mated with FLP recombinase-expressing mice to remove the neo-cassette, and their offspring were mated to Cre recombinase-expressing mice to delete *Slc44a2* exons 3–10.

129/SvJ mouse strain genomic DNA was obtained from the Jackson Laboratory (Bar Harbor, ME). Three pairs of primers (Table 1) were used to amplify the *Slc44a2* targeted region (exons 3–10) (Fig. 1A) and the 5′ and 3′ homology arms on either side of these exons. The 5′ homologous (5′ HA) arm spans 3.1 kb of the distal end of intron 2; the 1.9-kb targeted deletion site (TDS) includes exons 3–10; and the 3′ homologous arm (3′ HA) spans the 2.7-kb proximal region of intron 10. The PCR products were cloned into pGEM-T vectors, and wild-type sequences were verified by the University of Michigan DNA Sequencing Core. Standard molecular cloning techniques were used to insert the *Slc44a2* fragments into the pLoxPFlpNeo vector (Hiraoka et al. 2006). This vector contains the neomycin resistance gene (*PGKneo* cassette) between FRT recombination sites for antibiotic selection after transfection of the targeting construct into mouse embryonic stem (ES) cells. The final targeting construct contained the TDS between the loxP recombination sites, the 5′ HA preceding the 5′ FRT site and the 3′ HA following the 3′ loxP site (Fig. 1B).

**TABLE 1:** Primers used for cloning the targeting region

5′ KO F	TTCCTTGCTCTGCTTTGTAAGTCC	5′ KO R	CTGTTCATTTTGAAAACTTT-	3.1 kb
Ex KO F	AGACCAGACCTGGTGGCACAG	Ex KO R	GGTAGCAGACATTTGGCACTT	1.9 kb
3′ KO F	CCGGGACCGCTTTCAGAA	3′ KO R	GACATTAGGTAAACATCTTCA	2.7 kb

5′ KO F and 5′ KO R amplify the 5′ homologous region; Ex-KO F and Ex KO R amplify the exon 3–10 targeted deletion site; and 3′ KO F and 3′ KO R amplify the 3′ homologous region

The targeting vector was linearized with SacII and electroporated into R1 agouti mouse ES cells (129X1/SvJx129S1/SvJ) (Nagy et al. 1993). Electroporation and antibiotic selection of mouse ES cells were performed at the University of Michigan Transgenic Animal Model Core (Hughes and Saunders 2011). Genomic DNA prepared from *neo*^*R*^ ES cell clones was analyzed for homologous recombination of the targeting construct by long-range genomic PCR (Expand Long Kit—Roche, Indianapolis, IN) with two primer sets (Table 2): GGGGGCAGGGAGGGCTAAATCT (forward, Hour F (Homologous upstream region Forward)) located upstream of the 5′ HA and GCCTACCGGTGGATGTGGAATGTG (reverse, Hour R) derived from the *PGKneo* cassette, and with control primers for the wild-type *Slc44a2* gene: GCACCGAAGGAATGGGGAAGGAT (nHourF (normal HourF)) located upstream of the 5′ HA and CATCTCGCCAGCCCCAGGTCATAC (PosR-nHourF (Positive control reverse primer for nHourF)) located near the distal end of the 5′ HA (Fig. 1B).

**TABLE 2:** Sequencing the rCTL2 insert from clone 29 (E29/plate)

Hour F	GGGGGCAGGGAGGGCTAAATCT	Hour R	GCCTACCGGTGGATGTGGAATGTG
nHour F	GCACCGAAGGAATGGGGAAGGAT	PosR-nHourF	CATCTCGCCAGCCCCAGGTCATAC
5′ HO F	CCCAAAGATCCAAAACAGTCCACA	5′ HO R	GCCCGGGCTACATTGAGAGTC
5′ HO F1	TTTATGTCTGTGGTCTGCGTTGTG	5′ HO R1	CAGCCAAACCTTATCTCCAGTCCT
Lox P scrn F	GGCAGGACAGCAAGGGGGAGGAT	Lox P scrn R	ACGGGGCTCTGGGTCTCAAAGTT
Ex KO F	GGGCGAATTCTGTGGGCAAAAG	Ex KO R	AGGGGAAGAGGTGAAACGCATTAC
3′ KO F	CCGGGACCGCTTTCAGAA	3′ KO R	GACATTAGGTAAACATCTTCA
3′ HO R	GCCGGGGCTACATTGAGAAACT	3′ HO R1	GTAGAGCCGCAAACGCAGACAGTA
3′ KO-3′ HO F1	GCAGGTCGAGGGACCTAATAAC	3′ KO-3′ HO R1	GGTGTTAGCAGGCCTGGAGAAATG
3′ HO F2	TCAGTTCCAGCTTTCAGTTTCCTT	3′ HO R2	AGCCACAACCATAGCACCTCACTG
3′ HO F3	GGTCTGCCCCCTTCGTTTTACA	3′ HO R3	ACGGGGCTCTGGGTCTCAAAGTT
3′ HO F4	GGCCGTCTGAGCTTGATGTCTGTC	3′ HO R4	AATTATGCACAGGAGGCTTAGTCA
3′ HO F7	CCTGGCTTTTCTGTATTCTGATGC		
3′ HO F	GGCGACTTGATATTTGTTTTTGAT		
3′ HO F5	CCTGGATGGCTTTTAGTGAGTTGC		

### Confirmation of Recombinant Clones Using Southern Blot

A 411-bp hybridization probe (probe 1) was amplified from genomic DNA sequences located upstream of the 5′ HA, gel purified, doubly radiolabelled with ^32^P-dCTP plus ^32^P-dATP (Perkin Elmer, Waltham, MA) using the Prime-Gene Labeling System (Promega, Madison, WI), and separated from unincorporated nucleotides by centrifugation through a Sephadex column. Ten micrograms of genomic DNA from PCR-positive ES clones was digested overnight with HindIII in the presence of 4 mM spermidine. Restriction fragments were separated by electrophoresis on 0.8 % agarose gels with 1× TBE buffer and transferred to Zetaprobe GT filters (Bio-Rad Labs, Hercules, CA). The radiolabeled probe was prehybridized with 50 μg mouse Cot-1 DNA (Life Technologies, Invitrogen, Grand Island, NY) at 65 °C 30 min before adding the probe to the filters. Hybridization with the radiolabeled probe was carried out in 0.5 M sodium phosphate and 7 % SDS, pH 7.2, at 65 °C overnight. Filters were washed at 65 °C for 15 min twice in the following solutions containing 0.1 % SDS: 2× SSC, 1× SSC, 0.2× SSC, and 0.1× SSC. Biomax-MS film (Kodak) was exposed to filters with an intensifying screen at −80 °C for 2 days (Meisler et al. 2013).

### Sequencing of ES Cell Clone 29, Subcloning, Selection, and Blastocyst Injection and Implantation

DNA was isolated from 1500 *neoR* colonies of electroporated ES cells and examined for proper insertion of the construct. PCR analysis determined a single clone (clone 29) was positive for correct homologous recombination. The construct from positive clone 29 was completely sequenced to ensure an intact insertion. The full set of sequencing primers is given in Table 2. Clone 29 was found to be heterogeneous and was subcloned; 50 subclones were rescreened by genotyping (primers in Table 3). PCR-positive subclones with normal karyotypes were selected for blastocyst injection and implantation in pseudopregnant mice. Male progeny (ES cell-mouse chimeras) were selected based on high coat color contribution and genotyping results and were bred to wild-type C57BL/6 mice to generate N1 offspring. N1 germ line founders carrying the *Slc44a2*^*neo*^ allele (*B6;129Sv-Slc44a2*^*tm1Tec*^) were crossed with pCAGGs-FLP mice (Kranz et al. 2010). The resulting N2 *Slc44a2*^*flox*^ mice (*B6;129Sv-Slc44a2*^*tm1.1Tec*^) were then crossed with B6.FVB-Tg(EIIa-Cre) mice carrying a Cre recombinase gene on the adenovirus EIIa promoter (Cat Number 003724) (Jackson Labs, Bar Harbor, ME) to delete *Slc44a2* exons 3–10 and generate the mutant *Slc44a2*^Δ^ allele (*B6;129Sv-Slc44a2*^*tm1.2Tec*^).

**TABLE 3:** Primers used for genotyping

Primer name	Sequence
NeoGenoF2	TGA TGC TCT TCG TCC AGA TCA TCC
NeoGeno R1	TGA ACA AGA TGG ATT GCA CGC AGG
NeoDelFn2	GGACTCGGTGCCCTTCTGGA
NeoDelRn2	TGGCTGGCCTAAAACCTGCTATG
Exon D F1	GGGGATGCGGTGGGCTCTAT
Exon D R1	AGCCACAACCATAGCACCTCACTG
ExonD F2	TCATGTGAGTTGGAAGGAAAGTGG
ExonD R2	AGTCACTGGGCTAAAGAGATGTC
ExonD F	GGGGATGCGGTGGGCTCTAT
ExonD R	TTGGTCGGTTAAACAGTGGAGAA
NeoExoD F	TTTGCCTGGTGAATGCTGAGAGTA
NeoExoD R	TTGGTCGGTTAAACAGTGGAGAA

### DNA/RNA Extraction, Genotyping, RT-PCR, and Sequencing

To obtain genomic DNA, mouse tail tip biopsies were each digested overnight at 55 °C in 1 ml of tail solubilization buffer (TSB) (Miller et al. 1988) containing 45 μl (>27 AU) proteinase K (Qiagen, Valencia, CA; cat 19131) activated at 37 °C. TSB as modified here consisted of 100 mM Tris-HCl pH 8.5, 5 mM EDTA, 0.2 % SDS, and 200 mM NaCl. After digestion, the samples were cooled and 400 μl of chilled tail salts (4.21 M NaCl, 0.63 M KCl, and 10 mM Tris (pH 8.0)) was added to precipitate the proteins and SDS. The samples were vigorously mixed and incubated for 1 h at 4 °C. Insoluble material was removed by centrifugation and genomic DNA was precipitated from the supernatant by the addition of two volumes of cold 100 % ethanol, and centrifugation. After washing the pellet, the extracted DNA was dissolved in 150 μl TE buffer (Invitrogen, Grand Island, NY) and then PCR-genotyped. Both Expand Long Template PCR and GC Rich kits (Roche Diagnostics, Indianapolis, IN) were used with Hour primers (Table 2). Genotyping primers used to assess deletion of the neo-cassette and the exons 3–10 are listed in Table 3. Cochlea (one to two ears), lung, and kidney tissues (~30 mg) were homogenized using a 1.5-ml disposable pellet/pestle tube or a polytron homogenizer, and RNA was isolated using RNeasy mini kit or a Trizol/chloroform method (Qiagen, Valencia, CA). Reverse transcription PCR (RT-PCR) was performed (Kommareddi et al. 2010), and complementary DNA (cDNA) was prepared from total RNA isolated from homozygous knockout FVB (lung) and C57BL/6 (kidney) tissues; these were analyzed for *Slc44a2* expression and submitted for Sanger sequencing using the primers shown in Table 4.

**TABLE 4:** Primers used for Slc44a2 full-length cDNA sequencing of wild-type and knockout mice

Mouse-specific full-length primers for isoforms P1 and P2	
MsP1 CTL2/SLC44A2 F	TCGCGCTGGCTTCGGACTCA
MsP1 CTL2/SLC44A2 R	GCACAGGGCTGGGCATA CAAG
MsP2 CTL2/SLC44A2 F	GCGGGTGGCGGCTGTGTC
MsP2 CTL2/SLC44A2 R	GGATGGCCAAAGTAGGGGTGAGG
Sequencing primers	
Forward primers	Location and name
1. ATGGGACGCCTCAGAAATACGA	Ms AK041533 90–111 bp MsCTL2F1
2. ACACTCGCGACTTTGACTATTACA	Ms AK041533 438–461 bp MsCTL2F2
3. GCGATTCCTGGCTGGCATTAT	Ms AK041533 814–834 bp MsCTL2F3
4. CCCCTTGCGGAATGAGAGC	Ms AK041533 1294–1312 bp MsCTL2F4
5. GGTACCACACGGGCTCCTTAG	Ms AK041533 1551–1571 bp MsCTL2F5
6. ATTGTGGGTAGTGTGGGCATCC	Ms AK041533 1880–1901 bp MsCTL2F6
Reverse primers	Location and name
1. AGAAGCTTGCTGCAGACATCCTA	Ms AK041533 2472–2494 bp MsCTL2R1
2. GTCAGGATCGGAACCCAGTAATAA	Ms AK041533 1963–1986 bp MsCTL2R2
3. GCCCGTGTGGTACCTGAGTGCT	AK041533 1543–1564 bp MsCTL2R3
4. CCCTGCTGGCTTCTTTGATG	Ms AK041533 1093–1112 bp MsCTL2R4
5. AAAGGTTTGCTGGGGGTGATA	Ms AK041533 538–558 bp MsCTL2R5
6. CAGGGGGTTGGCACACTTCA	Ms AK041533 278–297 bp MsCTL2R5

### Backcrossing to the FVB Strain

B6;129Sv-*+/Slc44a2*^Δ^ mice carrying the targeted allele were backcrossed to FVB/NJ mice for seven generations (N7) to generate a line (*FVB.129Sv-Slc44a2*^*tm1.2Tec*^) with 99 % FVB genetic background on average. Intercrosses were carried out at N7 to assess hearing loss in F1 wild-type (*+/+*), heterozygous (*+/Slc44a2*^Δ^), and homozygous mutant (*Slc44a2*^Δ/Δ^) progeny. Heterozygous progeny mice were intercrossed to maintain the strain. Representative mice from this N7-F1 group were used for immunohistochemistry and morphometric analysis, auditory brain stem response (ABR) testing, spiral ganglion cell counts, and necropsy.

### Immunohistochemistry and Morphometric Analysis

Cochleae from FVB N7-F1 +/+, *+/Slc44a2*^Δ^, and *Slc44a2*^Δ/Δ^ mutant mice were processed for whole-mount immunohistochemistry and morphometric analysis. Immunohistochemistry was done using anti-myosin 7A (hair cells) and rhodamine phalloidin (actin) using published protocols (Sha et al. 2008). Expression of Slc44a2 in the transgenic mice was carried out using affinity-purified rabbit anti-CTL2-NT as described previously (Beyer et al. 2011). Inner ear cross sections were generated in the KHRI Core Histology Facility and evaluated. Photographs were taken using both light and confocal microscopy. Hair cell counts were completed from surface preparations as described (Sha et al. 2008). The cytocochleogram-obtained data was compared to a database of normal CBA/J mouse cochlea, and missing hair cell percentages were calculated for each row and plotted as a function of the distance from the apical to the basal end of the surface preparation.

### Auditory Brain Stem Response Testing and Analysis

ABR testing (Sha et al. 2008) was carried out at 12, 24, and 32 kHz at different time points. Mixed models were generated to examine differences in hearing thresholds among the three genotype groups while accounting for age, frequency, and repeated measures on the same mice. Comparisons focused on relating FVB N7-F1 *+/Slc44a2*^Δ^ and *Slc44a2*^Δ/Δ^ mice to *+/+* mice. Kaplan-Meier curves were also produced to demonstrate the time to development of threshold >50 dB sound pressure level (SPL) by genotype. A 50-dB SPL was selected since it represents the start of moderate hearing loss. Statistical significance was defined as *p* < 0.05. Mixed model and Kaplan-Meier analyses were performed using Stata 13.1 (StataCorp, 2013 StataCorp, College Station, TX).

### Spiral Ganglion Cell Counts and Analysis

Counts of spiral ganglion cell (SGC) bodies in apical, middle, and basal turns from FVB N7-F1 mice were performed using published methodology (Sha et al. 2008). Raw counts and spiral ganglion cell density (cells/10,000 μm^2^) were determined and then analyzed. A generalized linear model (GLM) examined the role of cochlear location (apex, middle, base) and genotype (i.e., +/+, *+/Slc44a2*^Δ^, *Slc44a2*^Δ/Δ^) as a correlate of SGC density. Statistical significance was defined as *p* < 0.05. The Tukey-Kramer method was used to adjust *p* values for multiple pairwise comparisons. GLM and post hoc comparisons were performed using SAS 9.4 (SAS Institute, 2014, Cary, NC).

### Full Phenotype Analysis (Necropsy)

FVB N7-F1 mice were analyzed for whole-body histopathology in the Unit for Laboratory Animal Medicine, Pathology Service. Animals were euthanized humanely using CO_2_, and euthanasia was confirmed with thoracotomy. Full necropsies were performed on animals in the Unit for Laboratory Medicine Pathology sections by MH. This evaluation included complete blood counts and assessments of brain, heart, lung, liver, kidney, spleen, mesenteric lymph node, thymus, eye, skin, skeletal muscle, stomach, duodenum, jejunum, ileum, colon, rectum, and reproductive tract, which were collected in 10 % neutral buffered formalin (NBF) for routine histopathology examination. The tympanic bullae were opened with a 22-gauge needle and placed in 10 % NBF for 48 h. The heads were then sectioned at the posterior aspect of the eyes to remove the rostral portion of the skull, and sectioned along the midline. The soft tissue around the tympanic bullae was dissected away, and specimens were placed in Immunocal (Decal Corporation, Tallman, NY) for 24 h for decalcification. Specimens were embedded in paraffin and sectioned routinely for histopathology.

### Immunoprecipitation and Western Blot

CTL2-NT antibody raised in rabbits immunized with an SCL44A2 unique but phylogenetically conserved antigenic peptide encoded by exon 2 in the N-terminal portion of SLC44A2 was used for immunoprecipitation and Western blot analysis of SLC44A2 in lung and kidney extracts prepared as described previously (Nair et al. 2004; Kommareddi et al. 2009, 2010).

## Results

### Generation of a Targeted Deletion of *Slc44a2*

**FIG. 2:**
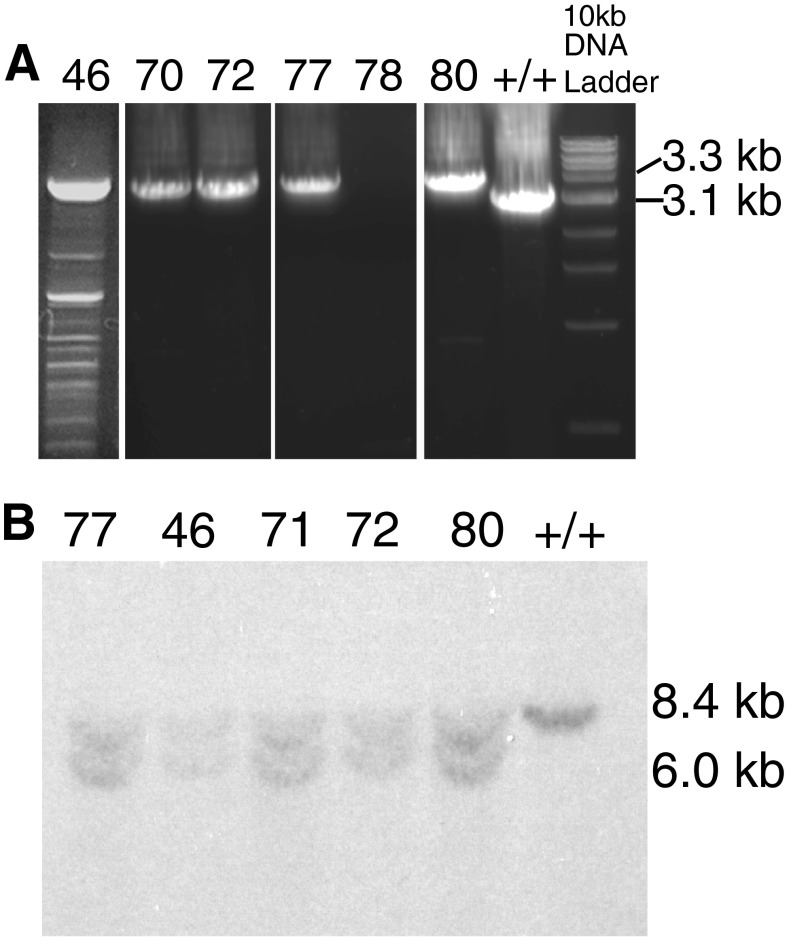
PCR and Southern blot results for five positive ES cell subclones. **A** PCR genotyping of DNA from the five positive ES cell subclones carrying the *Slc44a2* targeting construct (3.3-kb PCR product), subclone 78 that lacked the targeting construct, and +/+ (wild-type) mouse DNA (3.1-kb PCR product) without the construct. **B** Southern blot showing the presence of the *Slc44a2* 8.4-kb wild-type and the 6.0-kb recombinant construct in the five positive subclones of clone 29.

The *Slc44a2/Ctl2* gene has 22 exons encoding 706 amino acids. Exons 3–10 encode the first and largest extracellular loop of the protein (Nair et al. 2004) shown in green in Figure 1A. The KHRI-3 antibody that induces hearing loss in mice and guinea pigs (Nair et al. 1995, 1997) targets an N-linked carbohydrate moiety of the SLC44A2 protein (Kommareddi et al. 2010). Two of the consensus N-linked glycosylation sites (shown as open triangles, Fig. 1A) are located on the first outer loop (Nair et al. 2004; Kommareddi et al. 2010). Based on the logic that this large protein loop is important to the normal function of the protein, exons 3–10 were chosen for deletion in ES cells using a targeting vector (Fig. 1B). Correct targeting in ES cell clones was verified by PCR and Southern blot analysis. One ES cell clone, number 29, was targeted correctly. This clone was subcloned and progeny clones were PCR tested for the 3.3-kb recombined insert containing the neo-cassette (Figs. 1B and 2A). Incorrectly targeted clones showed no 3.3-kb band (clone 78) and wild-type cells (+/+) had only the 3.1-kb band. The murine *Slc44a2* gene has two *Hind*III sites, one upstream of the 5′ homologous region and the other in the 3′ homologous region, which span 8.4 kb in length in the wild-type mouse. After recombination, a new *Hind*III site in the vector located after the 5′ FRT site (Fig. 1B) was expected to produce a 6.0-kb 5′ fragment in *Hind*III-digested DNA of correctly targeted clones. Southern blot analysis demonstrated that this 6.0-kb fragment (as well as the wild-type 8.4-kb fragment) was recognized by the probe in five PCR-positive subclones (Fig. 2B).

**FIG. 3:**
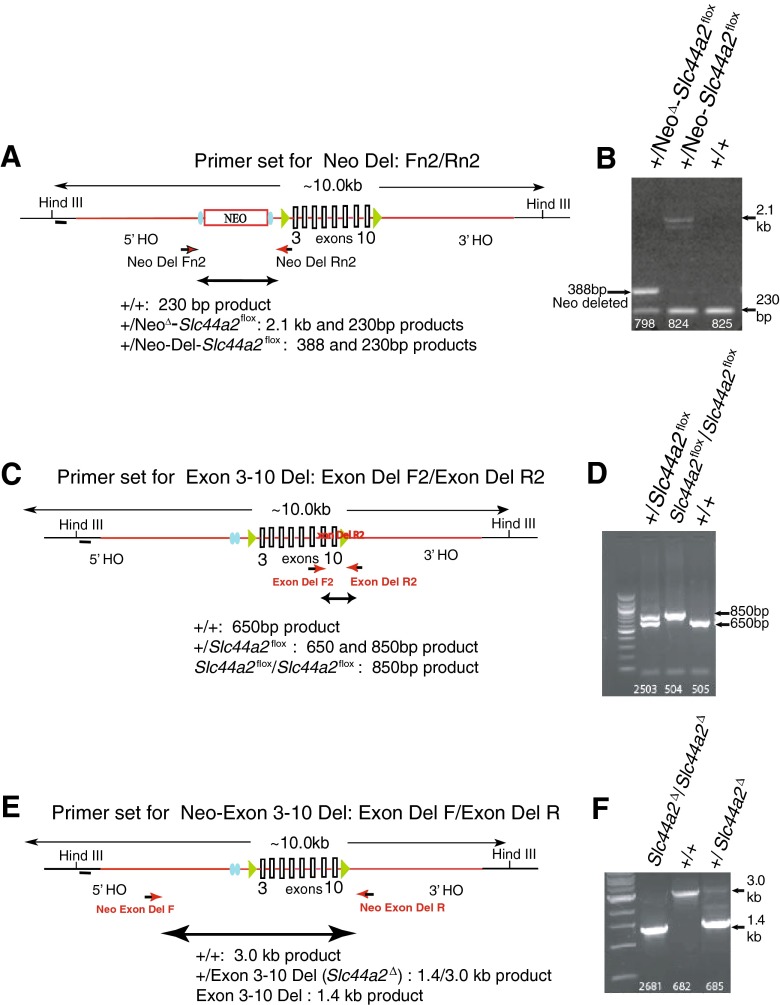
Deletion schema in transgenic mice showing the primer sets and PCR results used to demonstrate successful deletion of the neo-cassette and *Slc44a2* exons 3–10. **A**, **B** PCR results for heterozygous +/neo-deleted floxed *Slc44a2*, +/*Neo-Slc44A2*, and a wild-type animal +/+ without the construct. **C**, **D** PCR results for correct location of floxed exons 3–10 in heterozygous +/*Slc44a2* ^*flox*^ and homozygous *Slc44a2* ^*flox*^/*Slc44a2* ^*flox*^, as well as +/+ mice. **E**, **F** PCR results for homozygous *Slc44a2* ^Δ^/*Slc44a2* ^Δ^, heterozygous +/*Slc44a2* ^Δ^, and +/+ mice after crossing to C57BL/6 carrying Cre.

The five correctly targeted ES subclones (Fig. 2A, B) were sequenced and verified to contain the complete *Slc44a2* recombinant gene. Three clones exhibiting normal karyotypes were injected into blastocysts and implanted in pseudopregnant female mice. ES cell-mouse chimeras were bred to C57BL/6 mice. Germ line transmission of the transgene from all three clones was detected in genomic DNA of resulting N1 offspring using primer sets that amplify across the neo-cassette and exons 3–10. Transgene-positive mice were crossed with C57BL/6 mice carrying FLP recombinase (Kranz et al. 2010), resulting in progeny mice (*Neo*^Δ^*Slc44a2*^*flox*^) with successful removal of the neo-cassette (Fig. 3A, B). Neo-deleted *Slc44a2*^*flox*^/+ mice were intercrossed to obtain progeny that were +/+, +/*Slc44a2*^*flox*^, and *Slc44a2*^*flox*^/*Slc44a2*^*flox*^ (Fig. 3C, D). Homozygous *Slc44a2*^*flox*^/*Slc44a2*^*flox*^ mice were crossed with C57BL/6 mice carrying Cre recombinase to produce the mutant *Slc44a2*^Δ^ allele with deletion of exons 3–10. The +/*Slc44a2*^Δ^ mice were then crossed and the progeny were found to have the expected distribution of +/+, +/*Slc44a2*^Δ^, and *Slc44a2*^Δ/Δ^ genotypes (Fig. 3E, F).

### Mutant *Slc44a2*^Δ/Δ^ Mice on the C57BL/6 Background

**Figure 4:**
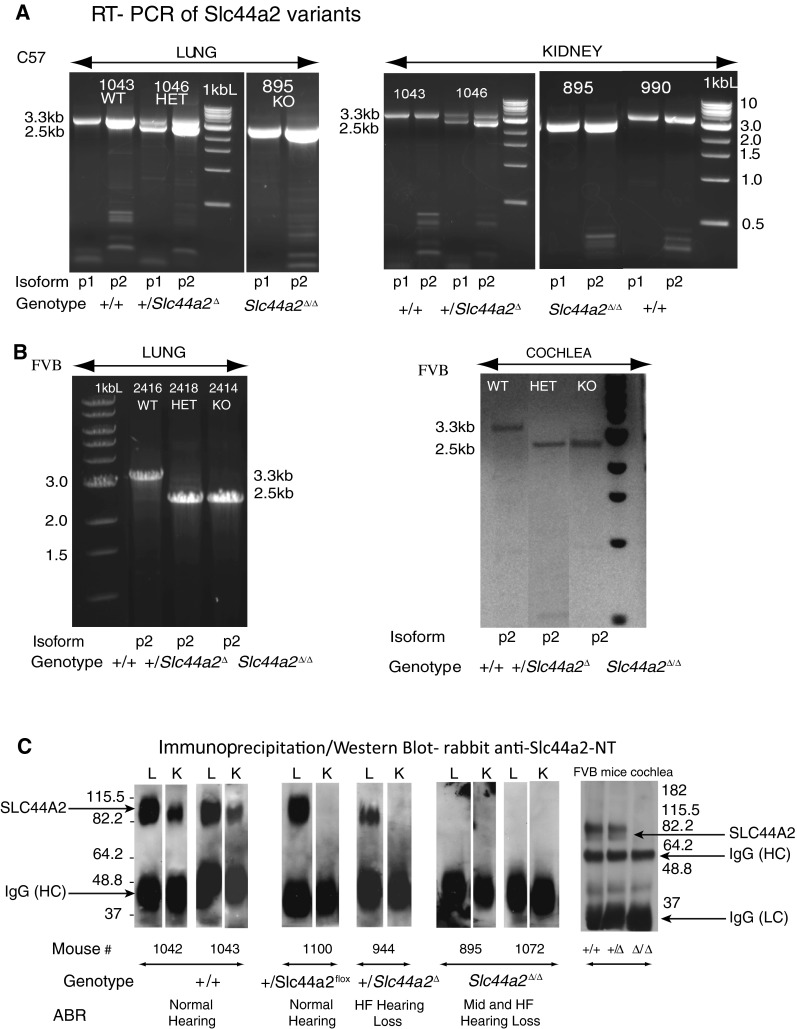
Expression of Slc44a2 message and protein in wild-type and knockout mice. **A** RT-PCR using lung and kidney mRNA in the C57BL/6 strain. The figure demonstrates expression of the mRNA for both the P1 and P2 *Slc44a2* isoforms. Wild-type mouse 1043 and 990 (*left and right panels*) expressed the full-length 3.3-kb cDNA; heterozygous mouse 1046 (+/*Slc44a2* ^Δ^) (*center panels*) expressed both the full-length 3.3-kb and the truncated 2.5-kb message; and homozygous knockout mouse 895 (*Slc44a2* ^Δ/Δ^) (*right panels*) expressed only the truncated 2.5-kb message. In lung, the P2 isoform is more abundant than P1; however, in kidney, they are equally expressed. **B** RT-PCR showing expression of the Slc44a2 P2 isoform in lung and cochlea in WT, +/+, HET, +/*Slc44a2* ^Δ^, and KO *Slc44a2* ^Δ/Δ^ FVB mice. In FVB mice, the overall expression of the Slc44a2 message is less strong than that in the C57 strain. In FVB heterozygous mice lung and cochlea, the full-length wild-type message was not detected due to competition by the shorter deleted transcript. To make the PCR product in FVB cochlear extract more visible, the photograph of the gel was reversed to black on white. **C** Immunoprecipitation and Western blot results using rabbit anti-SLC44A2-NT. Slc44a2 expression is shown in lung and kidney samples of C57BL/6 +/+, +/*Slc44a2* ^flox^, +/*Slc44a2* ^Δ^, and *Slc44a2* ^Δ/Δ^ littermates with different genotypes. Mice 1042 and 1043 (+/+) strongly express Slc44a2 in lung (*L*) and to a lesser extent in kidney (*K*) and have normal hearing. Slc44a2 is expressed in the lung of heterozygous mouse 1100 carrying one wild-type allele and one recombinant allele (+/*Slc44a2* ^*flox*^, exons 3–10 not yet deleted), but expression is below the limit of detection in kidney tissue. Mouse 1100 has normal hearing. Heterozygous mouse 944 (+/*Slc44a2* ^Δ^, neo; exons 3–10 deleted) has low expression of Slc44a2 in lung and below detectable levels in kidney. This mouse has hearing loss at high frequency—65-dB SPL threshold at 48 kHz. Homozygous knockout mice 895 and 1072 (*Slc44a2* ^Δ/Δ^) have no Slc44a2 expression in lung or kidney. Mouse 895 has 50- and 70-dB SPL thresholds at 24 and 48 kHz. Mouse 1072 had a 65-dB SPL threshold at 48 kHz. Repeated tests using 12 % gels to detect the 150-amino acid SLC44A2^Δ/Δ^ fusion protein encoded by the alternate transcript showed no expression of the 17-kDa protein (not shown). Analysis of protein extracts of cochlea from FVB mice from all three genotypes show the same results as in C57BL/6 mice with protein expression in wild-type and heterozygous mice but not in knockout mouse (*right panel*). Note that the protein is expressed in the +/Slc44a2^Δ^ mice even though the message was competed for by the deleted message in the RT-PCR shown in Figure 4B.

*Slc44a2*^Δ/Δ^ mice appeared healthy and fertile and did not exhibit circling or head tilting behavior. No visible size difference or other atypical observations were noted upon necropsy between normal and knockout mice except for changes in the inner ear described below. RT-PCR of RNA from lung and kidney tissue of +/+, +/*Slc44a2*^Δ^, and *Slc44a2*^Δ/Δ^ mice (C57BL/6 background) was conducted to detect the expression of *Slc44a2* isoforms P1 and P2 (P1, using the upstream exon 1a, and P2, using the more proximal exon 1b) (Kommareddi et al. 2010). Full-length transcripts (3.3 kb) of both isoforms were expressed in +/+ mice. Both isoforms of the 3.3-kb full-length transcripts and both isoforms of the deleted 2.5-kb *Slc44A2*^Δ^ transcript (lacking exons 3–10) were expressed in the +/*Slc44a2*^Δ^ animals. As expected in *Slc44a2*^Δ/Δ^ mutants, only the 2.5-kb *Slc44A2*^Δ^ transcripts of the P1 and P2 isoforms were observed (Fig. 4A). Similarly, after transfer of the deleted allele to the FVB strain (Fig. 4B), the wild-type 3.3-kb transcript was produced in +/+ mice, but only the deleted 2.5-kb transcript could be amplified in cDNA from the lung and cochlea of the +/*Slc44a2*^Δ^ and *Slc44a2*^Δ/Δ^ mice (Fig. 4B).

Immunoprecipitation of the Slc44a2 protein from lung and kidney tissue of +/+, +/*Slc44a2*^Δ^, and *Slc44a2*^Δ/Δ^ mice was performed using a rabbit SLC44A2-NT antiserum. (This antiserum, formerly called CTL2-NT, for the N-terminal CTL2 domain, was raised to the peptide encoded by exon 2 (Nair et al. 2004; Kommareddi et al. 2009).) The immunoprecipitated proteins were separated by gel electrophoresis and Western blot analysis was performed using the SLC44A2-NT antiserum. The full-length SLC44A2 protein was prominent in lung and to a lesser extent in kidney tissue from +/+ mice. Heterozygous +/*Slc44a2*^Δ^ mice and heterozygous mice carrying the recombinant +/SLC44A2^flox^ allele (neo-deleted but exons 3–10 intact) exhibited reduced protein expression in lung and undetectable protein in kidney. Since the kidney expresses much lower levels of SLC44A2 than lung (Kommareddi et al. 2010), the absence of one allele apparently was sufficient to reduce expression in kidney to below detectable levels. No protein was expressed in the *Slc44a2*^Δ/Δ^ mutants (Fig. 4C). Similar results were observed in protein extracts from the cochlea of FVB mice after the transfer of the deleted allele to this strain (Fig. 4C, right panel). Note that the wild-type protein band in cochlear extracts of the FVB +/*Slc44a2*^Δ^ mice is expressed. The RT-PCR failed to detect the wild-type cDNA in the heterozygous mice (Fig. 4B) due to relatively low transcript expression and PCR competition by the shorter deleted transcript.

**FIG. 5:**
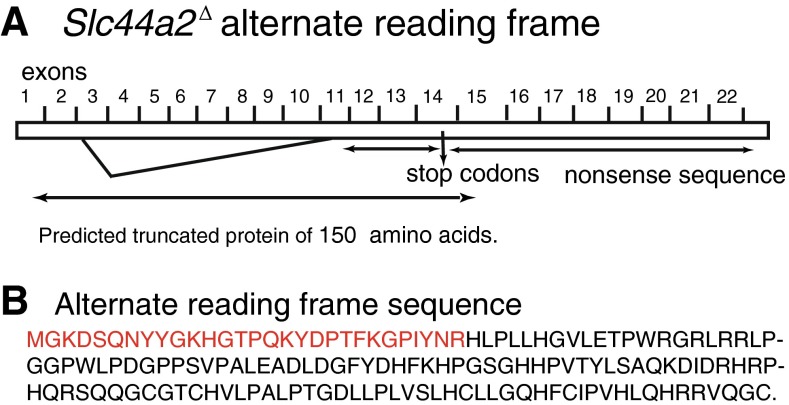
**A** Structure of the deleted *Slc44a2* gene. The open reading frame of this mutant transcript matched the wild-type *Slc44a2* transcript through exon 2 and the first amino acid of exon 3, but exhibited a frameshift in exon 11 that encoded alternative residues until a stop codon within exon 14. **B** Putative 150-amino acid truncated protein deduced from the RT-PCR expressed message. The first 29 amino acids shown in *red* are identical to the sequence encoded by exons 1–2 and the first amino acid of exon 3. The open reading frame is spliced into exon 11 out-of-frame resulting in an alternate transcript (*in black*) that fails to match to any known proteins. If the protein was produced and stable, it would have a molecular mass of 17 kDa. As the N-terminal amino acids are conserved in the fusion transcript, immunoprecipitation and detection with the N-terminal antibody should detect the fusion protein if it is produced and stable.

Sequencing of the 2.5-kb transcript *Slc44a2*^Δ/Δ^ mice revealed splicing from exon 2 into exon 11 (Fig. 5A, B). The open reading frame of this mutant transcript matched the wild-type *Slc44a2* transcript from exon 1 through exon 2 including the first amino acid encoded by exon 3. However, a single nucleotide frameshift within exon 11 resulted in an alternative reading frame that lacked similarity to Slc44a2 or any known protein (Fig. 5B). The reading frame was terminated by a stop codon within exon 14. The putative truncated protein, if stable, is predicted to be 150 amino acids in length (17 kDa), with the first 29 amino acids identical to intact Slc44a2 (red text, Fig. 5B). Multiple attempts to detect the predicted truncated protein on 12 % gels to insure visualization of low-mw proteins failed to detect the fusion protein in either *Slc44a2*^Δ/Δ^ or +/*Slc44a2*^Δ^ mice (data not shown), indicating that the fusion protein is either not made or is unstable.

### Mutant *Slc44a2*^Δ/Δ^ Mice Exhibit High-Frequency Hearing Loss, Loss of Hair Cells, and Spiral Ganglion Degeneration

At 3 months of age, ABR analysis of *Slc44a2*^Δ/Δ^ mice on the C57BL/6J genetic background revealed poor hearing thresholds of 65–90 dB SPL at 48 kHz and 50–70 dB SPL at 24 kHz associated with outer hair cell losses in the basal turns of the cochlea (data not shown). In contrast, the +/+ and *+/Slc44a2*^Δ^ mice exhibited thresholds of 20–30 dB SPL at these frequencies and relatively low levels of hair cell loss in the basal turn (data not shown). C57BL/6 mice carry the *Cdh23*^*753A*^ allele at the *Ahl* locus that is associated with ARHL (Noben-Trauth et al. 2003). To avoid the additional effects of this allele on hearing, we backcrossed *Slc44a2*^Δ^ mice to the FVB/NJ strain, which carries the wild-type *Cdh23*^*753G*^ allele associated with lower sound thresholds in later life (Noben-Trauth et al. 2003). N2 backcross mice carrying the *Slc44a2*^Δ^ allele and homozygous for the FVB *Cdh23*^*753G*^ wild-type allele were used as breeders for subsequent FVB backcrosses leading to the N7 generation. At backcross generation N7 (99 % FVB genetic background), heterozygous *+/Slc44a2*^Δ^ mice were intercrossed to produce F1 wild-type, heterozygous, and knockout animals at the *Slc44a2* locus. Knockout *Slc44a2*^Δ/Δ^ mice in the FVB/NJ background were generally smaller than their wild-type littermates but were otherwise healthy and fertile (data not shown). Anatomic pathology review of systems by one of us (MH) found no abnormalities of any organ system (other than the inner ear), including lung and kidney which strongly express Slc44a2.

**FIG. 6:**
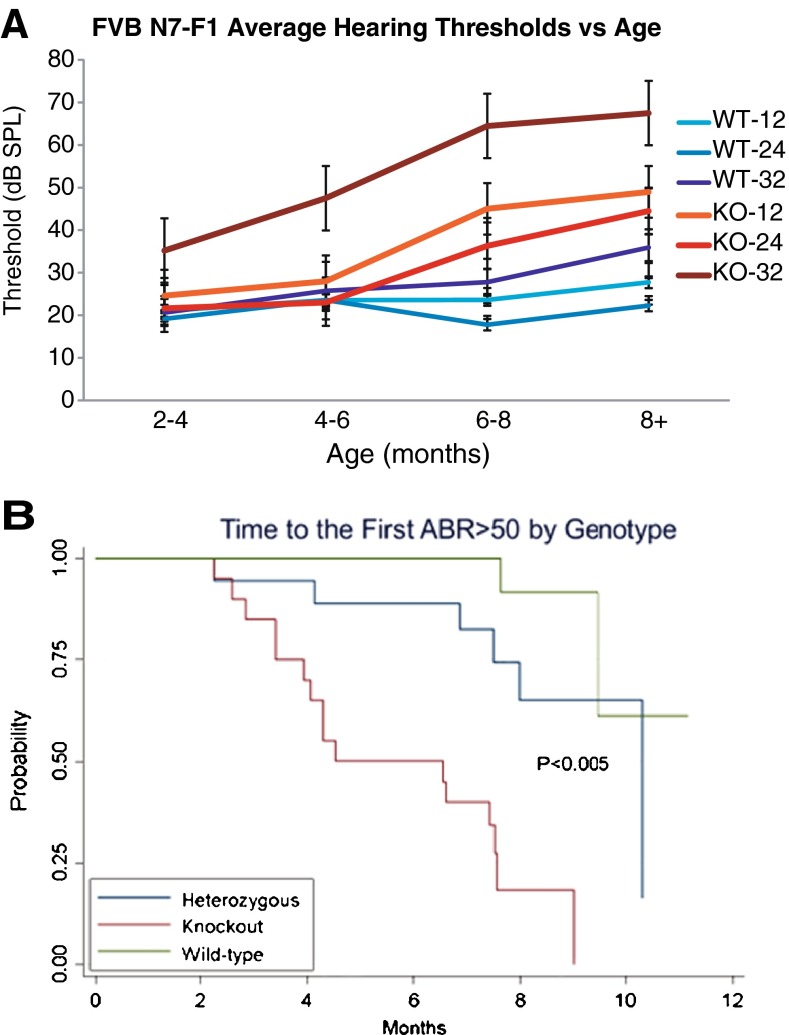
*Slc44a2* ^Δ/Δ^ mice on the FVB background exhibit progressive hearing loss. A Mice carrying the wild-type (*WT*, +/+) and the homozygous knockout (*KO*, *Slc44a2* ^Δ/Δ^) *Slc44a2* alleles on an FVB/NJ background (N7-F1) were evaluated longitudinally for hearing at 2–4 months of age to 8 or more months. ABRs to pure tone stimuli at 12, 24, and 32 kHz were measured periodically on the mice as they aged. Each pure tone frequency is indicated by a *different shade of color* (12 kHz—*light*, 24 kHz—*medium*, 32 kHz—*dark*). Mean threshold responses are indicated for WT (*blue*) and KO (*red*) mice; *error bars* represent standard deviation. *N* = 20 for WT and *n* = 20 for KO, but some were studied histologically during the course of the follow-up, resulting in different numbers of mice at the later time points. At all frequencies, WT mice maintained relatively normal hearing over time with threshold means between 20 and 25 dB SPL. Mutant KO mice demonstrated progressive hearing loss at all frequencies. *Slc44a2* loss has the greatest effect at high frequency, whereas hearing loss was milder at 12 and 24 kHz. **B** Kaplan-Meier curve presenting the likelihood of an animal from each genotype group first developing a hearing threshold above 50 dB SPL (moderate hearing loss). This curve accounts for all three testing frequencies (12, 24, and 32 kHz). Each *line* shows the probability of mice in each group *not* yet having a threshold greater than 50 dB SPL. The curves decline over time, as the probability for first developing a threshold >50 dB SPL increases with age.

Fifty-nine F1 intercross progeny from FVB/NJ backcross generation N7 (20 +/+, 19 *+/Slc44a2*^Δ^, and 20 *Slc44a2*^Δ/Δ^) were tested for hearing using ABR analysis in response to 12, 24, and 32 kHz pure tone stimuli. In Figure 6A, average hearing thresholds calculated from repeated measures of the same mice as they aged from 2 to more than 8 months are shown for the +/+ and Δ/Δ groups. In *Slc44a2*^Δ/Δ^ mutants, hearing loss at 32 kHz developed early and became notably progressive at all frequencies with age. At all three frequencies, the hearing thresholds, adjusted for age, were significantly worse in the *Slc44a2*^Δ/Δ^ mutants than in either the +/+ or +/*Slc44a2*^Δ^ mice (*p* < 0.0001). Heterozygous +/*Slc44a2*^Δ^ mice exhibited progressive hearing loss at 32 kHz from 4 to >8 months of age (not shown). In contrast, +/+ (all frequencies) and +/*Slc44a2*^Δ^ mice (12 and 24 kHz) exhibited minimal threshold shifts with aging (≤15 dB). At 24 kHz, changes in threshold after age adjustment were not significantly different between +/+ and +/*Slc44a2*^Δ^ mice (*p* values >0.3). A full model, which included all variables, further demonstrated that ABR results in +/+ and +/*Slc44a2*^Δ^ mice did not significantly differ (*p* values >0.17) after adjustment for age and frequency. Kaplan-Meier curves demonstrate time to development of first threshold >50 dB SPL by genotype (Fig. 6B). We used 50 dB SPL since this represents moderate hearing loss and there were not enough mice who reached the >80-dB SPL level to warrant analyses. Figure 6B shows the results regardless of testing frequency and demonstrates significantly increased and faster development of hearing loss with age among the knockout mice compared to the wild-type and heterozygous groups (*p* = 0.005).

**FIG. 7:**
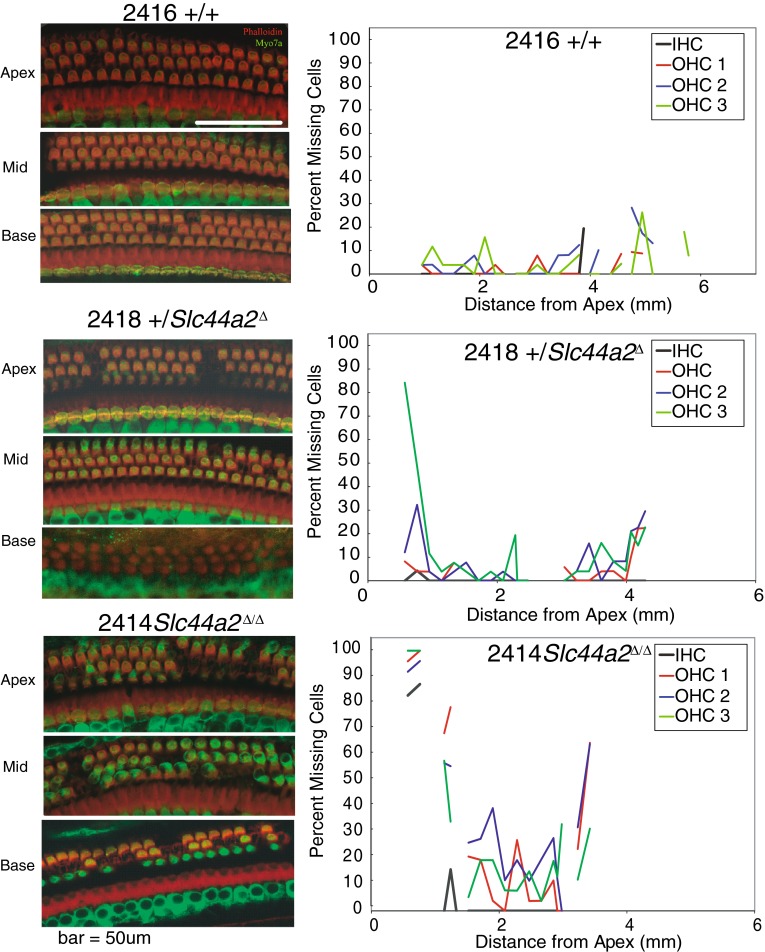
Whole-mount surface preparations of the inner ear from FVB N7 F1 mice at 8 months of age. *Left panels*: indirect immunofluorescence (*red*—phalloidin and *green*—myosin 7a). *Right panels*: cytocochleograms of wild-type mouse 2416 (+/+), heterozygous 2418 (+/*Slc44a2* ^Δ^), and knockout 2414 (*Slc44a2* ^Δ/Δ^). *Top panels*: +/+ mouse 2416 surface preparation and cytocochleogram display minimum loss of hair cells from apex to base. *Middle panels*: +/*Slc44a2* ^Δ^ mouse 2418 exhibits more frequent outer hair cell loss in the apex and basal turns, and cytocochleogram displays the same pattern (depicts a “U” shaped loss, a typical pattern of age-related loss/damage). *Bottom panels*: mouse 2414 (*Slc44a2* ^Δ/Δ^) demonstrates extensive hair cell loss in all three turns.

**FIG. 8:**
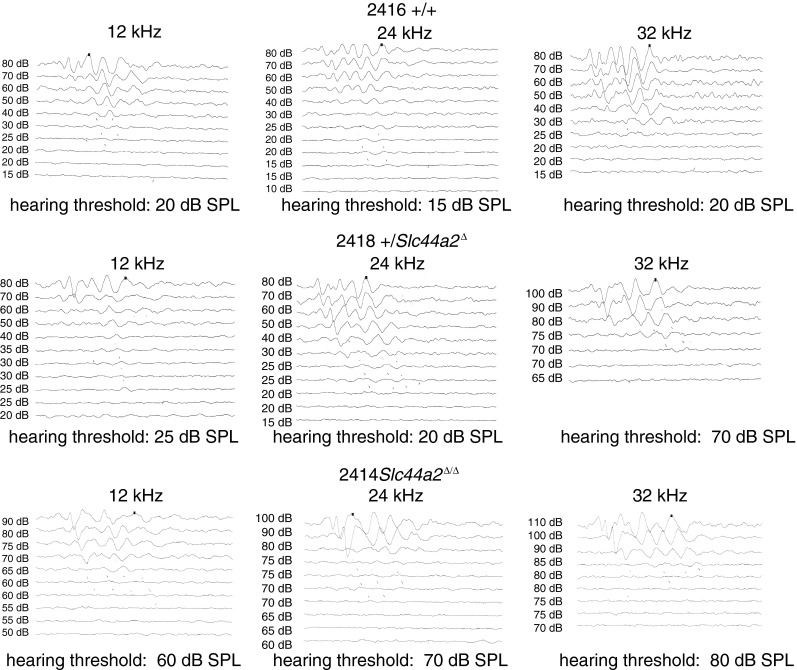
ABR recordings from the same mice presented in Figure 7. *Top row*: mouse 2416 (+/+) exhibits normal hearing at all three frequencies. *Middle row*: mouse 2332 (+/*Slc44a2* ^Δ^) exhibits an elevated threshold at 32 kHz but normal hearing thresholds at 12 and 24 kHz. *Bottom row*: mouse 2414 (*Slc44a2* ^Δ/Δ^) exhibits elevated thresholds at all three frequencies consistent with the frequent hair cell loss and spiral ganglion loss as shown in Figure 9.

**FIG. 9:**
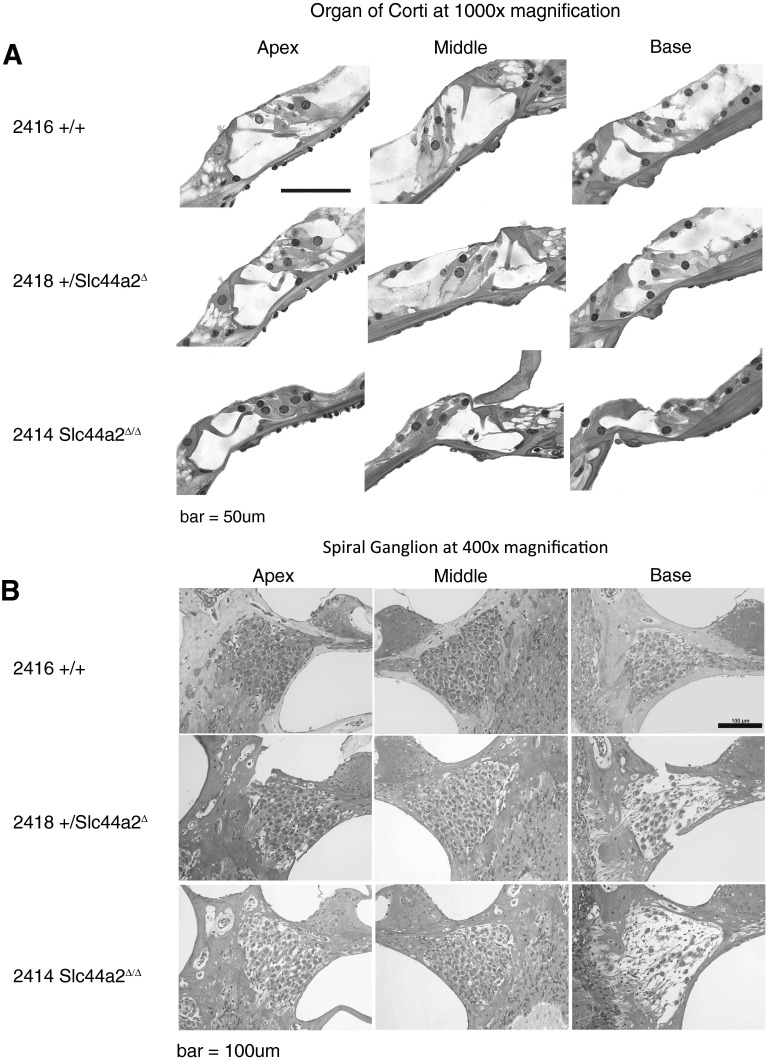
Mid-modiolar cochlear plastic sections of the opposite ear of the same FVB N7 F1 transgenic mice shown in Figure 7. **A** *Top panels* show sections through the organ of Corti of +/+ mouse 2416, +/*Slc44a2* ^Δ^ mouse 2418, and *Slc44a2* ^Δ/Δ^ mouse 2414. The organ of Corti appears relatively normal in these sections in +/+ and +/*Slc44a2* ^Δ^ mice. However, in the *Slc44a2* ^Δ/Δ^ mouse, the organ of Corti in the base appears abnormal with no detectable tunnel of Corti and has poor representation of both inner and outer hair cells. **B** The *lower panels* depict the spiral ganglion from the same sections as shown in the upper panels. Note that in both the +/*Slc44a2* ^Δ^ and *Slc44a2* ^Δ/Δ^ mice, the spiral ganglion in the basal turn exhibits loss of numerous cell bodies.

**FIG. 10:**
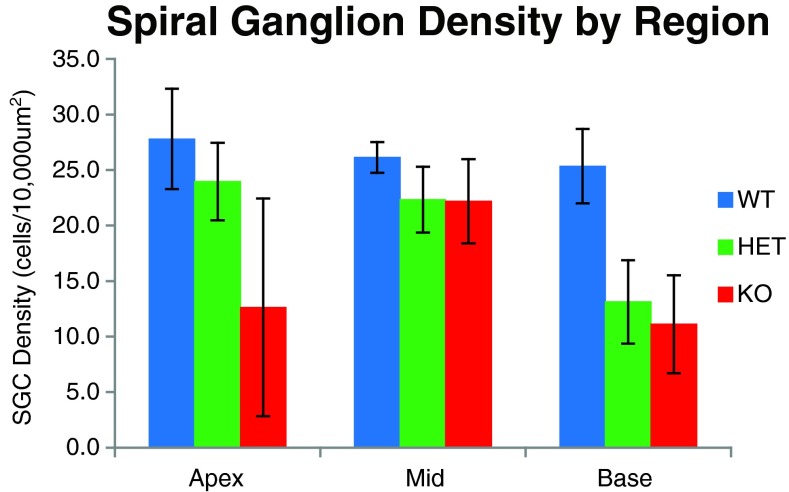
Spiral ganglion cell density (cells/10,000 μm^2^) across the apex, middle, and basal turns of the cochlea for 5 +/+, 4 +/*Slc44a2* ^Δ^, and 7 *Slc44a2* ^Δ/Δ^ animals. SGCs were intact for +/+ mice at all regions. The apical and basal parts of the spiral ganglion of *Slc44a2* ^Δ/Δ^ mice showed extensive loss of cells, while +/ *Slc44a2* ^Δ^ mice demonstrated density loss only at the base.

Histologic examination of the inner ear was carried out on a randomly selected subset of these 59 mice (*N* = 25) after their final ABR to correlate hearing status with cochlear morphology. Surface preparations of the organ of Corti were evaluated for hair cell survival. Representative images from organ of Corti surface preparations and cytocochleograms from FVB N7-F1 +/+, +/*Slc44a2*^Δ^, and mutant *Slc44a2*^Δ/Δ^ mice are shown in Figure 7, together with the ABR recordings for these three mice (Fig. 8). By 8 months of age, the high hearing thresholds observed in *Slc44a2*^Δ/Δ^ mutants across all measured frequencies are associated with relatively high levels of loss of both inner and outer hair cells throughout the cochlea. With the exception of the extreme base and apex of the cochlea, hair cell loss at 8–9 months is relatively minimal in +/+ and +/*Slc44a2*^Δ^ mice, which exhibit lower sound thresholds. The opposite ear of each of the representative animals was decalcified, fixed, and sectioned to examine the organ of Corti and the spiral ganglion in mid-modiolar sections (Fig. 9). The mid-modiolar sections through the organ of Corti were consistent with the surface preparation data. The organ of Corti has a normal appearance in all three turns of the +/+ mouse. The apex and middle turns of the mouse exhibited near-normal appearance of the organ of Corti, but in the basal turn, the outer hair cells were missing. Visible stereocilia can be seen in some sections corresponding to both inner and outer hair cells in the +/+ and +/*Slc44a2*^Δ^ mice. The *Slc44a2*^Δ/Δ^ mouse organ of Corti has a near-normal appearance in the apex and middle turns, but in the basal turn, the morphology is abnormal with absence of the tunnel of Corti and no distinguishable hair cells (Fig. 9A). These observations bear similarity to the results in Figure 7. Further examination of the mid-modiolar sections also revealed gross abnormalities of the spiral ganglion cells in the basal turn of the +/*Slc44a2*^Δ^ mouse and the Δ/Δ mouse. Multiple mid-modiolar sections from 5 +/+, 4 +/*Slc44a2*^Δ^, and 7 *Slc44a2*^Δ/Δ^ mice were examined for spiral ganglion cell density as previously described (Sha et al. 2008). The results are depicted graphically in Figure 10. The basal segment of the spiral ganglion of the +/*Slc44a2*^Δ^ and *Slc44a2*^Δ/Δ^ animals displayed extensive loss of cells although severity varied from animal to animal. In contrast, spiral ganglion cells were intact in the +/+ mice (Fig. 9B). A generalized linear model that examined both cochlear location (*p* = 0.0026) and genotype (*p* < 0.0001) revealed that these two are independently and significantly associated with spiral ganglion cell density (Table 5). SGC density is different between the base and middle turns (*p* = 0.0017); the differences between the base and apex (*p* = 0.1080) and middle and apex (*p* = 0.2456) are not statistically significant. When comparing density across genotypes, there is a statistically significant difference between +/+ and +/*Slc44a2*^Δ^ (*p* = 0.0114) and +/+ and *Slc44a2*^Δ/Δ^ (*p* < 0.0001). The difference between heterozygous and knockout mice approaches significance (*p* = 0.0834) (Table 5).

**TABLE 5:** SGC analysis of location/genotype vs. density

Source	DF	Type III SS		Mean square	*F* value	Pr > F	
Location	2	438.701667		6.87	0.0026	219.350833	
Genotype	2	1080.297071		16.91	<0.0001	540.148536	
LS means: location vs. density				LS means: genotype vs. density			
Pr > |t| for H0: LSMean(*i*) = LSMean(*j*)				Pr > |t| for H0: LSMean(*i*) = LSMean(*j*)			
*i*/*j*	Base	Mid	Apex	*i*/*j*	WT	Het	KO
Base		0.0017	0.108	WT		0.0114	<0.0001
Mid	0.0017		0.2456	HET	0.0114		0.0834
Apex	0.108	0.2456		KO	<0.0001	0.0834	

Analysis of the role of cochlear location (apex, middle, base) and genotype (WT [+/+], HET, [+/*Slc44a2* ^Δ^], KO [*Slc44a2* ^Δ/Δ^]) as a correlate of spiral ganglion cell density. Statistical significance defined as *p* < 0.05. When comparing density vs. location, SGC density is only different between the base and middle turns (*p* = 0.0017). For genotype vs. density, a statistically significant difference was found between WT and HET (*p* = 0.0114) and WT and KO (*p* < 0.0001). The difference in SGC density between HET and KO mice approaches significance (*p* = 0.0834)

**FIG. 11:**
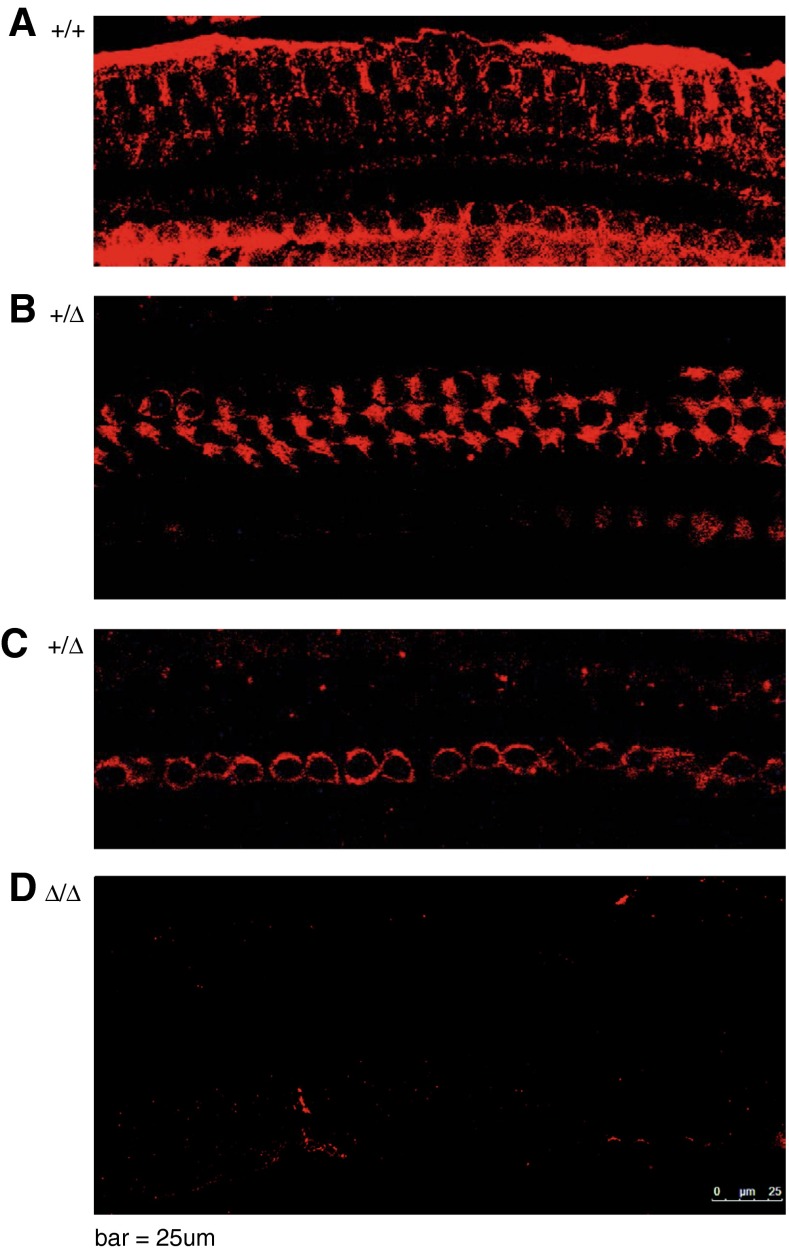
Whole-mount surface preparations of the inner ear from FVB N7 F4 mice labeled with affinity-purified rabbit anti-SLC44A2 antibody (CTL2-NT) (*red*). FVB wild-type (+/+) (*upper panel*) and heterozygous (+/*Slc44a2* ^Δ^) (*middle panels*) exhibit respectively high and moderate levels of Slc44a2 protein expression in supporting cells in the organ of Corti; *Slc44a2* ^Δ/Δ^ mice fail to express Slc44a2 (*lower panel*) in the organ of Corti.

Inner ear surface preparations from other ++/, +/*Slc44a2*^Δ^, and mutant *Slc44a2*^Δ/Δ^ mice were also stained by indirect immunofluorescence with affinity-purified rabbit anti-Slc44a2 (CTL2-NT) as shown in Figure 11. Note that the expected expression of Slc44a2 on supporting cells is present in +/+ (Fig. 11A), less so in +/slc44a2^Δ^ (Fig. 11B, C), and completely absent from *Slc44a2*^Δ/Δ^ ears (Fig. 11D), which is consistent with the Western blots shown in Figure 4C.

## Discussion

A full understanding of the roles of supporting cells in maintenance of inner ear function is only beginning (May et al. 2013; Monzack and Cunningham 2013; Wan et al. 2013). Similarly, the role of the supporting cell protein SLC44A2 in inner ear function is not well understood. SLC44A2 was discovered as the antigenic target of a monoclonal antibody (KHRI-3) raised against isolated cells from the inner ear (Zajic et al. 1991). KHRI-3 showed strong reactivity with supporting cells, but not sensory cells, and the antibody target was called the “supporting cell antigen.” We demonstrated upregulation of KHRI-3 antigen in areas of hair cell loss induced by drugs or noise (Ptok et al. 1993). Subsequent studies revealed that systemic KHRI-3 antibody was capable of causing hearing loss in mice (Nair et al. 1995). Infusion of purified KHRI-3 into the guinea pig cochlea led to antibody binding to supporting cells and loss of sensory hair cells. Where hair cells were missing, KHRI-3 antibody staining intensity of adjacent supporting cells was increased (Nair et al. 1997). Additionally, potent antigen expression occurred where adjacent supporting cell membranes form scars in the space left by the dying hair cell (Nair et al. 1997). These findings strongly suggested that the KHRI-3-defined supporting cell antigen has important functions in survival of sensory cells in the cochlea, and that antibody binding to supporting cells interferes with this function leading to hair cell death. Additionally, the accumulation of the protein at the scars implies a role for SLC44A2 in maintaining the integrity of the reticular lamina of the organ of Corti.

Immunoprecipitation of the supporting cell antigen from guinea pig inner ear extracts using KHRI-3 yielded protein bands of 68 and 72 kDa. The peptides from the trypsin-digested bands identified by tandem mass spectroscopy when compared to known proteins (Nair et al. 2004) showed homology to the newly identified CTL1 (choline transporter-like protein 1) (O’Regan et al. 2000) and identity to its putative homolog CTL2 (choline transporter-like protein 2) (Nair et al. 2004). The choline transporter-like proteins (CTL1–CTL5) were later classified as members of the superfamily of solute carrier (SLC) proteins, and CTL2 was designated SLC44A2. Recombinant CTL1 (SLC44A1) functions in choline transport in several vertebrate models (O’Regan et al. 2000; Michel and Bakovic 2012). *SLC44A2* has more homology to *SLC44A4* and *SLC44A5* than to *SLC44A1* and *SLC44A3* (Traiffort et al. 2013). However, the function of SLC44A2 remains uncertain. Using transduced CHO cells, we demonstrated only low-level transport of choline by the SLC44A2-P2 isoform preferentially expressed in the murine inner ear (Beyer et al. 2011), but not by the SLC44A2-P1 isoform that is preferentially expressed in human and guinea pig inner ear (Kommareddi et al. 2010). However, two groups have implicated choline transport by SLC44A2/CTL2 in human cancer cells. Nakamura et al. observed glucocorticoid-induced expression of *CTL1* and *CTL2* messenger RNA (mRNA) and increased uptake of tritiated choline in the human lung cancer cell line A549. siRNA knockdown of *CTL1* or *CTL2* mRNA specifically decreased CTL1 or CTL2 expression and choline uptake (Nakamura et al. 2010). Similarly, knockdown of *CTL1*, *CTL2*, or *CTL5* decreased choline transport in H82 small cell lung cancer cells (Song et al. 2013). Membership of CTL2/SLC44A2 in the solute carrier protein family strongly suggests a role in transporting a charged molecule across the membrane of inner ear supporting cells. The knockdown experiments also suggest that these members of the CTL/SLC44 family may work together to form a functional transporter system, since knockdown of any one of the three members of the family decreases choline transport in human cancer cells. This may explain why expressing SLC44A2 alone was not efficient in inducing choline transport in CHO cells.

SLC44A2 is strongly expressed in supporting cells in the vestibular system (Nair et al. 2004; Beyer et al. 2011) and in the developing mouse otic capsule. In our developmental study (Beyer et al. 2011), SLC44A2 was expressed in hair cells and supporting cells at embryonic days E17 and E18, but expression in hair cells declined and remained only in supporting cells by postnatal day P1. Therefore, this protein also may have a role in the early development of the inner ear.

In the current study, we report the effects on hearing of a targeted deletion of exons 3–10 of *Slc44a2* in the mouse. We opted to delete exons 3–10 that encode the first and largest outer loop of the Slc44a2 molecule. The first outer loop has two N-glycosylation sites that likely contribute to SLC44A2 function, since the KHRI-3 antibody binds to the N-linked carbohydrate, but not the protein backbone of SLC44A2 (Nair et al. 2004). Studies of human antibodies to the human neutrophil antigen HNA3a carried on the SLC44A2 molecule (Curtis et al. 2010; Greinacher et al. 2010; Bux 2011) also suggest an important role for the first outer loop. SLC44A2 is strongly expressed in lung (Kommareddi et al. 2010) and on subsets of human white blood cells, notably granulocytes (Curtis et al. 2010; Greinacher et al. 2010; Bux 2011). The HNA3a/HNA3b neutrophil antigens are defined by a polymorphism at codon 152 of the *SLC44A2* gene that encodes either arginine or glutamine at this site. HNA3a antibodies arise in individuals typed as HNA3b and bind to SLC44A2 molecules that express arginine 152. Such antibodies agglutinate granulocytes and cause degranulation, indicating a key functional significance of the SLC44A2 first outer loop. Antibody binding to this molecule on granulocytes causes degranulation and release of cytokines resulting in acute lung injury (Curtis et al. 2011). Antibody binding to lung tissue itself (Kommareddi et al. 2010) may also contribute to antibody-mediated lung damage in transfusion-related lung injury (Bux 2011). Furthermore, KHRI-3 binding to SLC44A2 interferes with a supporting cell function that is essential for hair cell survival, since hair cells begin to die and hearing loss ensues after antibody binding (Nair et al. 1995, 1997, 1999).

We developed the targeted *Slc44a2*^Δ^ allele in the C57BL/6 mouse strain. *Slc44a2* transcripts expressed from the Δ allele spliced out of frame from exon 2 to exon 11, resulting in a shift of reading frame and a subsequent premature stop codon. Mice homozygous for the deletion allele (*Slc44a2*^Δ/Δ^) lacked detectable expression of the full-length SLC44A2 protein. There was no expression of a truncated protein encoded by the aberrantly spliced Δ allele, suggesting that this mutation is likely null. After the *Slc44a2*^Δ^ allele was transferred to the FVB/NJ genetic background to remove the mutant *Cdh23*^*753A*^ allele carried by C57BL/6J, progressive high-frequency hearing loss at 32 kHz developed as early as 4 months in the homozygous *Slc44a2*^Δ/Δ^ mice. Progressive hearing loss also developed in these mice at 12 and 24 kHz after 6 months of age. Hearing loss in the FVB *Slc44a2*^Δ/Δ^ mice is accompanied by extensive outer hair cell loss as well as patchy inner hair cell loss and loss of spiral ganglion cells in the basal turn were found in both the *Slc44a2*^Δ/Δ^ mice and in some *+/Slc44a2*^Δ^ mice, suggesting that loss of even one copy of the gene has adverse effects on inner ear homeostasis.

This study has shown the effects of *Slc44a2* deletion on hearing in a knockout mouse. Further study is necessary to determine the mechanism underlying the effects of deletion of *Slc44a2* on hair cell survival and hearing. SLC44A2 is strongly expressed in supporting cells (Deiter’s cells, pillar cells, inner border cells, and inner phalangeal cells) directly adjacent to inner and outer hair cells, both of which are lost to various degrees in *Slc44a2*^Δ/Δ^ mutants (Nair et al. 1997, 1999; Kommareddi et al. 2007). SLC44A2-enriched supporting cells such as inner pillar cells, inner border cells, and the third row of Deiter’s cells are LGR5 positive and can act as progenitors for hair cells after damage (Shi et al. 2013; Bramhall et al. 2014). The role of SLC44A2 in supporting cell regeneration (Mellado Lagarde et al. 2014) and supporting cell transdifferentiation into hair cells (Korrapati et al. 2013; Shi et al. 2013; Bramhall et al. 2014) is yet to be examined. Antibody binding to SLC44A2, aminoglycoside treatment, or noise exposure result in extensive outer hair cell damage with concomitant upregulation of SLC44A2 expression (Ptok et al. 1993). Scar formation and sealing of the area of hair cell loss are the natural processes of the repair of the organ of Corti by supporting cells (Raphael and Altschuler 1991; Raphael 2002; Taylor et al. 2012), and our results suggest involvement of the SLC44A2 protein in this process (Nair et al. 1999).

We observed progressive hearing loss and SGC loss in our *Slc44a2*^Δ/Δ^ mice. We assume that this occurs due to the ablation of normal protein function in supporting cells that maintain healthy hair cells and protect the SGCs. Cunningham’s group showed the ability of supporting cells to protect hair cells through upregulation of HSP-70 protein alone (May et al. 2013). Similarly, insulin-like growth factor 1 upregulation in inner ear also protects hair cells through supporting cell signaling (Yamamoto et al. 2014). Loss of SGCs in the *Slc44a2*^Δ/Δ^ mice and in some *+/Slc44a2*^Δ^ mice is another consequence of this mutant gene. Abnormalities of SGC had not been observed in prior experiments with antibody-induced hearing loss and hair cell loss (Nair et al. 1995, 1997, 1999); thus, the abnormal SGC phenotype in the heterozygous and Scl44a2-knockout mice was new. SLC44A2 expression can be appreciated in the spiral ganglion (Kommareddi et al. 2007), but this has not been extensively evaluated and it is unknown if it has a direct effect in maintaining SGCs. Genetic deletion of *Slc44a2* and impairment of SLC44A2 function by antibody binding to N-linked carbohydrate modifications of the mature protein both lead to damage of mature sensory cells, which do not express SLC44A2. Stankovic et al. suggested a neuregulin (NRG1)-erbB2-erbB3 crosstalk in the inner ear that is essential for SGC survival. When supporting cell erbB receptor signaling is lost, SGC neuregulins (NRGs) are also lost and SGC degeneration occurs (Stankovic et al. 2004; Monzack and Cunningham 2013). It is conceivable that loss of SLC44A2 expression in the *Slc44a2*^Δ/Δ^ mice could link to changes in erbB receptor and/or NRG signaling as proposed (Stankovic et al. 2004). In our previous studies, we have showed that Slc44a2 protein expression is upregulated in damaged hair cell areas, and in our *Slc44a2* knockout study, absence of this protein results in progressive hearing loss and SGC loss. It can be concluded that *Slc44a2* function in supporting cells is required for long-term hair cell survival, spiral ganglion cell survival, and overall maintenance of hearing.
